# 

**Published:** 1897-03-06

**Authors:** 


					THE HOSPITAL. ABSOLUTELY PURE, therefore BEST. Mauch 6tu, 1897.
CADBURY'S
COCOA
CADBURY'S
COCOA
-"S^aSilS;.e!'p?,U!"",'re!cm *> 1=1 <=> NO alkalies used.
>
Lasoovt Western Ihfiahart
*0. 045.:"Vol. XXI. LSeSS&S] ?axuuday, 1
[arch Otii, 18U7. [
"ulb2p.U376.ermS'J PRICE twopence.

				

